# Association Between Length of Residence in the U.S. and Insurance Coverage Within U.S. Chinese Older Adults

**DOI:** 10.1093/geroni/igab046.3663

**Published:** 2021-12-17

**Authors:** Natalie Tuseth, XinQi Dong, Stephanie Bergren, Michael Eng

**Affiliations:** 1 Rutgers, The State University of New Jersey, New Brunswick, New Jersey, United States; 2 Rutgers, New Brunswick, New Jersey, United States; 3 Rutgers University, New Brunswick, New Jersey, United States; 4 NYU Grossman School of Medicine, New York City, New York, United States

## Abstract

Barriers to affordable insurance may worsen disparities among underserved populations. Immigrants with <5 years of residence are not eligible for Medicare and Medicaid and are potentially without affordable alternatives. This study aims to look at the relationship between length of residence in the U.S. and insurance coverage within U.S. Chinese older adults ages 65+.This study used data from a representative sample of 2,365 community-dwelling U.S. Chinese older adults age 65+. The association between length in the U.S. (<5, 6-10, 10+) and insurance status was analyzed using chi squared test and logistic regressions. Within this sample, 58 (2.78%) participants had coverage outside Medicare and Medicaid, with 279 participants reporting no coverage. The vast majority of participants living in the U.S. <5 years had no insurance (81.48%). In a fully adjusted model, participants who were older and female were positively associated with having insurance coverage (OR:1.11 [1.07,1.15] and OR:1.29 [0.88,1.90]). Conversely, both living in the U.S. <5 years (OR:0.009 [0.006, 0.014]),and between 5-10 years (OR:1.20 [0.13,0.30]) were negatively associated with insurance coverage. When including coverage outside of Medicaid and Medicare, residence <5 years and 5-10 years were still negatively associated with insurance coverage ((OR:0.13 [0.009,0.02]), and (OR:0.19 [0.13,0.30])). Vulnerable populations such as older immigrants may not have access to insurance outside of public options, making a 5-year waiting period an additional barrier to quality health care.

